# Multilocus Sequence Typing and Antifungal Susceptibility of Vaginal and Non-vaginal *Candida glabrata* Isolates From China

**DOI:** 10.3389/fmicb.2022.808890

**Published:** 2022-03-17

**Authors:** Yisheng Chen, Yongqin Wu, Kaiyi Lulou, Dongting Yao, Chunmei Ying

**Affiliations:** ^1^Department of Clinical Laboratory, Obstetrics and Gynecology Hospital of Fudan University, Shanghai, China; ^2^Division of Life Sciences and Medicine, Department of Clinical Laboratory, The First Affiliated Hospital of USTC, University of Science and Technology of China, Hefei, China; ^3^Department of Laboratory Medicine, Longhua Hospital, Shanghai University of Traditional Chinese Medicine, Shanghai, China

**Keywords:** *Candida glabrata*, multilocus sequence typing (MLST), vulvovaginal candidiasis, antifungal susceptibility, fluconazole, cross-resistance, genotype diversity

## Abstract

*Candida glabrata* is a common cause of *Candida* infections. In our present study, we investigated the antifungal susceptibility and molecular epidemiology of vaginal and non-vaginal *C. glabrata* isolates. Seventy-six vaginal *C. glabrata* strains isolated from patients with vulvovaginal candidiasis and 57 non-vaginal *C. glabrata* isolates were collected at two hospitals in Shanghai, China. Antifungal susceptibility was examined using a broth microdilution method. Multilocus sequence typing was used for genotyping. Overall, 28 (21.1%), 28 (21.1%), and 29 (21.8%) *C. glabrata* isolates were resistant to fluconazole, itraconazole, and voriconazole, respectively. Briefly, 18 (23.7%), 18 (23.7%), and 19 (25%) vaginal strains were resistant to fluconazole, itraconazole, and voriconazole. While the resistance to these antifungals were all 17.5% (10/57) in non-vaginal strains. All isolates retained susceptibility to amphotericin B, and only four non-vaginal isolates were caspofungin resistant. Genotyping identified 17 ST patterns. In non-vaginal samples, the same genotypes appear as in the vaginal samples, except for one genotype (ST-182), while in the vaginal samples more genotypes appear (ST8, ST19, ST45, ST55, ST66, ST80, ST138, and ST17). The most common genotype was ST7 (81 strains), followed by ST10 (14 strains) and ST15 (11 strains). The majority of resistant phenotype strains (25/30, 83.3%) correlated to the predominant genotype (ST7), and the rest belonged to ST3 (2/30, 6.7%), ST10 (1/30, 3.3%), ST19 (1/30, 3.3%), and ST45 (1/30, 3.3%). Our survey revealed cross-resistance in vaginal and non-vaginal *C. glabrata* isolates. Moreover, there is no genotype associated with the resistance phenotype.

## Introduction

*Candida* infections can affect the organs with clinical manifestations and can lead to high rates of morbidity and mortality (Hassan et al., 2021). The number of *Candida* infections has progressively increased over the past decade (Cortegiani et al., 2018). Although *Candida albicans* remains the most common *Candida* species, the increasing numbers of non-*albicans Candida* (NAC) isolates have become concerning (Soulountsi et al., 2021). *Candida glabrata*, which accounts for approximately 15–25% of hospital-acquired fungal infections, is the second most common species causing candidiasis, followed by *Candida albicans*, in the United States and northwestern Europe (Hassan et al., 2021). *C. glabrata* is also the NAC yeast frequently responsible for causing vulvovaginal candidiasis (VVC), which is a common yeast infection in young women (Makanjuola et al., 2018). Due to its intrinsic resistance or low susceptibility to azoles, invasive *C. glabrata* infections cause approximately 40–60% morbidity and mortality (Timmermans et al., 2018). A study conducted in China showed that 12.2% of *C. glabrata* isolates are fluconazole resistant, and 17.8% exhibit resistance to voriconazole (Chen et al., 2018).

Molecular typing of local strains is important in epidemiological investigations and the prevention of dissemination. The purpose of genotyping is to track the source of infection, clarify the route of transmission, and identify outbreaks. Numerous publications have reported several methods for genotyping, including multilocus sequence typing (MLST), restriction fragment length polymorphism, pulsed-field gel electrophoresis (PFGE), and microsatellite analysis (Kiasat et al., 2019; Khalifa et al., 2020; Canela et al., 2021; Dikmen et al., 2021). Among these techniques, MLST, with its high reproducibility and reliability, is frequently used for strain phylogeny and epidemiology comparisons worldwide (Canela et al., 2021).

A study performed by Canela et al. (2021) showed lower genetic variability in *C. glabrata* obtained from a Brazilian hospital than in other *Candida* species. Using MLST, Boonsilp et al. (2021) found that *C. glabrata* isolated from a tertiary care hospital in Bangkok, Thailand, shows a low level of genetic diversity and that the most common genotype is ST55 (60%), followed by ST7 (20%), ST195 (10%), and ST199 (10%). However, Khalifa et al. (2020) have shown that ST7 is the most prevalent genotype among patients with *C. glabrata* infection (35%) and that this genotype is associated with high resistance rates in Japan. Currently, *C. glabrata* strains are generally isolated from blood, mouth, anal and fecal swabs, joint fluid, sputum, and other sources, while vaginal isolates constitute only a few percent of the strains isolated worldwide.

In our present study, we investigated the drug resistance and molecular characteristics of 76 vaginal *C. glabrata* strains and 57 non-vaginal strains isolated from two hospitals in Shanghai, China. We also investigated the correlation between strain genotype and susceptibility to antifungal agents.

## Materials and Methods

### Clinical Isolates and Identification

A total of 677 *C. glabrata* clinical strains were isolated from the vaginal samples of patients with VVC and concomitant diseases, such as polycystic ovary syndrome, recurrent miscarriage, human papillomavirus infections, and so on, at the Obstetrics and Gynecology Hospital of Fudan University from January 2018 to March 2021. Additionally, 254 strains were collected from non-vaginal samples obtained from patients with multiple chronic underlying conditions, like pneumonia, diabetes, sepsis, malignant tumors, heart disease, and kidney disease, in Longhua Hospital, Shanghai University of Traditional Chinese Medicine, also from January 2018 to March 2021. All clinical isolates were identified using matrix-assisted laser desorption/ionization time-of-flight mass spectrometry (Bruker, Karlsruhe, Germany). Clinical information on the isolates, including antifungal susceptibility, was obtained from clinical records. Among them, all the resistant isolates were selected, excluding the same strain isolated in a single patient. The susceptible isolates were randomly selected. Finally, 76 vaginal and 57 non-vaginal *C. glabrata* isolates were conducted for the follow-up study.

### Antifungal Susceptibility Testing

According to the clinical record, a yeast antimicrobial test strip (ATB FUNGUS 3) was used to identify the antifungal susceptibility of *C. glabrata* clinical isolates.

The selected 133 *C. glabrata* isolates were assessed *in vitro* for their susceptibility to antifungal agents using the broth microdilution method. The antifungal drugs used in our present study included fluconazole (FLC), itraconazole (ITC), voriconazole (VRC), amphotericin B (AMB), and caspofungin (CAS). Stock solutions of the drugs tested were all prepared in dimethyl sulfoxide (Sigma, United States). The drugs tested were serially diluted twofold in RPMI 1640 medium (Sigma, United States). The final concentrations were 0.25–128 mg/L for FLC, 0.0625–32 mg/L for ITC, 0.0625–32 mg/L for VRC, 0.0625–32 mg/L for AMB, and 0.0156–8 mg/L for CAS. A 100-μl aliquot of each drug dilution was added to individual wells in 96-well plates (Corning, United States) in columns 2–11. The well at column 1 was drug free and served as a control. Next, 100 μl of cells was added to each well at a final concentration of 103 cells/ml, except for column 12, to which 200 μl of RPMI 1640 medium was added as a negative control. The plates were incubated at 35°C for 24 h. The minimum inhibitory concentration (MIC) of each antifungal agent was defined as the lowest concentration of the antifungal drug inhibiting 100% (for AMB) or 50% (for the other antifungals) of yeast growth; the results of MIC assessment for each drug were determined visually after 24 h of contact using that drug. MIC_90_ was defined as the concentration at which 90% of the tested strains’ growth was inhibited. Antifungal susceptibility was assessed three times for each strain. *Candida parapsilosis* (ATCC 22019) and *Candida krusei* (ATCC 6258) were used as quality control strains. Breakpoints were interpreted according to the Clinical and Laboratory Standards Institute guidelines M60-Ed1.

### Multilocus Sequence Typing

Genomic DNA was extracted as described previously with minor modifications (Khalifa et al., 2020). Briefly, yeast cultures were incubated overnight, collected, and mixed with lysis buffer. After vigorous vortexing and boiling, each solution was purified using equal volumes of phenol–chloroform–isoamyl alcohol. Then, the supernatants were precipitated using isopropanol and washed using 70% ethanol. The obtained DNA was dried, suspended in sterile Tris-EDTA buffer, and preserved at –20°C. Genotyping of the 133 *C. glabrata* isolates was performed using MLST; six genes including *FKS*, *LEU*, *NMT*, *TRP*, *UGP*, and *URA* were used as housekeeping genes (Table 1; Yao et al., 2019). PCR was performed using PrimeSTAR^§^ HS DNA Polymerase (Takara Bio Inc.). Each PCR mixture (25 μl) contained 5 μl 5 × Prime STAR buffer (Mg^2+^ Plus), 2 μl dNTP mixture (2.5 mM each), 1 μl forward primer and 1 μl reverse primer, DNA template, and 0.25 μl PrimeSTAR HS DNA polymerase (2.5 U/μl). The conditions for the PCR reactions were as follows: initial denaturation at 98°C for 10 s, then 30 cycles of 10 s at 98°C, 10 s at 55°C, and 1 min at 72°C. Whole products were sequenced, and *C. glabrata* genotype was identified according to sequence typing at https://pubmlst.org/cglabrata/. An ST in this study was defined as strains with identical genotypes.

**TABLE 1:** Primers used for multilocus sequence typing.

Genes	Primers	Sequences
*FKS*	Forward	GTCAAATGCCACAACAACAACCT
	Reverse	AGCACTTCAGCAGCGTCTTCAG
*LEU*	Forward	TTTCTTGTATCCTCCCATTGTTCA
	Reverse	ATAGGTAAAGGTGGGTTGTGTTGC
*NMT*	Forward	GCCGGTGTGGTGTTGCCTGCTC
	Reverse	CGTTACTGCGGTGCTCGGTGTCG
*TRP*	Forward	AATTGTTCCAGCGTTTTTGT
	Reverse	GACCAGTCCAGCTCTTTCAC
*UGP*	Forward	TTTCAACACCGACAAGGACACAGA
	Reverse	TCGGACTTCACTAGCAGCAAATCA
*URA*	Forward	AGCGAATTGTTGAAGTTGGTTGA
	Reverse	AATTCGGTTGTAAGATGATGTTGC

### Statistical Analysis

The antifungal resistance of FLC, ITC, VRC, AMB, and CAS was calculated. SPSS 18.0 software (SPSS Inc., Chicago, IL, United States) was used for data analysis, and chi-square test was used for the categorical variables. All descriptive data were presented as mean (range), and the count data were described as proportions. *p* < 0.05 was considered statistically significant.

A dendrogram based on the results of our MLST assay and minimum spanning tree algorithm was generated using BioNumerics software (version 7.6, Applied Maths Inc.). A cluster analysis using unweighted pair group method with arithmetic means and advanced analysis using MST for categorical data were performed to define the genetic relatedness of the 133 *C. glabrata* isolates used in this study. The *C. glabrata* strains with a similarity exceeding 80% were considered clonally related.

## Results

### Clinical Characteristics

Of the 133 *C. glabrata* isolates included in the further study, 76 vaginal *C. glabrata* strains were isolated from female patients with VVC; the median age was 34.4 years old (range: 18–50). In total, 57 non-vaginal *C. glabrata* isolates were obtained from elderly patients with multiple chronic underlying conditions, like neurologic disease, diabetes, metabolic syndrome, malignant tumors, blood diseases, heart disease, kidney disease, and so on. Of these 57 patients, 45.6% (26/57) were female patients and 54.4% (31/57) were male patients, and the median patient age was 79.1 years (range: 57–94). These non-vaginal strains were isolated from clinical samples of urine (36.8%, 21/57), mouth swabs (33.3%, 19/57), sputum (19.3%, 11/57), blood (7.0%, 4/57), and fecal swabs (3.5%, 2/57). Data on individual clinical characteristics are presented in Supplementary Material.

### Antifungal Susceptibility Testing

According to ATB FUNGUS, 677 vaginal *C. glabrata* strains and 254 non-vaginal *C. glabrata* isolates retained susceptibility to amphotericin B; 4.1% (28/677), 7.4% (50/677), and 3.8% (26/677) of the 677 vaginal strains exhibited resistance to fluconazole, itraconazole, and voriconazole, respectively. None of the isolates were resistant to caspofungin. While the resistance rates were 5.9% (15/254), 4.3% (11/254), and 5.1% (13/254) in 254 non-vaginal *C. glabrata* isolates, 6 out of 254 (2.4%) isolates were resistant to caspofungin. Thus, the overall resistance rates to amphotericin B, fluconazole, itraconazole, voriconazole, and caspofungin were 0, 4.6% (43/931), 6.6% (61/931), 4.2% (39/931), and 0.9% (6/931), respectively.

Next, we determined the *in vitro* MICs of the five antifungals used to treat the 133 *C. glabrata* isolates included in this study (Table 2). Overall, the isolates retained susceptibility to amphotericin B. However, 28 out of the 133 *C. glabrata* isolates were resistant to fluconazole. Additionally, 28 and 29 isolates were resistant to itraconazole and voriconazole, respectively, and only 4 isolates were resistant to caspofungin, which belonged to the non-vaginal isolates. Overall, 18 vaginal and 10 non-vaginal strains were resistant to fluconazole.

**TABLE 2:** Summary of *in vitro* antifungal susceptibilities of 76 vaginal *C. glabrata* and 57 non-vaginal *C. glabrata* isolates by broth microdilution method.

Strains	Antifungals (resistant breakpoints)	Number of isolates		MIC		
			
		Susceptible	Resistant (%)	MIC range	MIC_50_	MIC_90_
Vaginal *C. glabrata* (*n* = 76)	AMB (> 2)	76	0	0.5–1	1	1
	FLC (≥ 64)	58	18 (23.7)	4–256	16	128
	ITC (> 2)	58	18 (23.7)	0.5–64	1	64
	VRC (> 1)	57	19 (25.0)	0.125–32	0.5	4
	CAS (≥ 0.5)	76	0	0.015–0.125	0.03	0.06
Non-vaginal *C. glabrata* (*n* = 57)	AMB (> 2)	57	0	0.5–1	1	1
	FLC (≥ 64)	47	10 (17.5)	2–256	16	128
	ITC (> 2)	47	10 (17.5)	0.5–64	2	64
	VRC (> 1)	47	10 (17.5)	0.06–16	0.25	4
	CAS (≥ 0.5)	53	4 (7.0)	0.015–0.5	0.06	0.25
Total (*N* = 133)	AMB (> 2)	133	0	0.5–1	1	1
	FLC (≥ 64)	105	28 (21.1)	2–256	16	128
	ITC (> 2)	105	28 (21.1)	0.5–64	2	64
	VRC (> 1)	104	29 (21.8)	0.06–32	0.25	4
	CAS (≥ 0.5)	4	4 (3.0)	0.015–0.5	0.06	0.06

In total, 103 isolates showed a susceptible phenotype for the antifungals examined in this study. According to the results obtained using the broth microdilution assay and the generated dendrogram, we divided the remaining 30 resistant *C. glabrata* isolates into five groups (G) as follows: group 1 (G1) contained four isolates with a resistant phenotype to the remaining antifungals (fluconazole, itraconazole, voriconazole, and caspofungin); group 2 (G2) contained 22 isolates with a resistant phenotype to fluconazole, itraconazole, and voriconazole; group 3 (G3) contained two isolates with a resistant phenotype to itraconazole and voriconazole; group 4 (G4) contained one isolate with a resistant phenotype to fluconazole and a resistant phenotype to voriconazole; and group 5 (G5) contained one isolate with a resistant phenotype to fluconazole.

### Multilocus Sequence Typing

**FIGURE 1:**
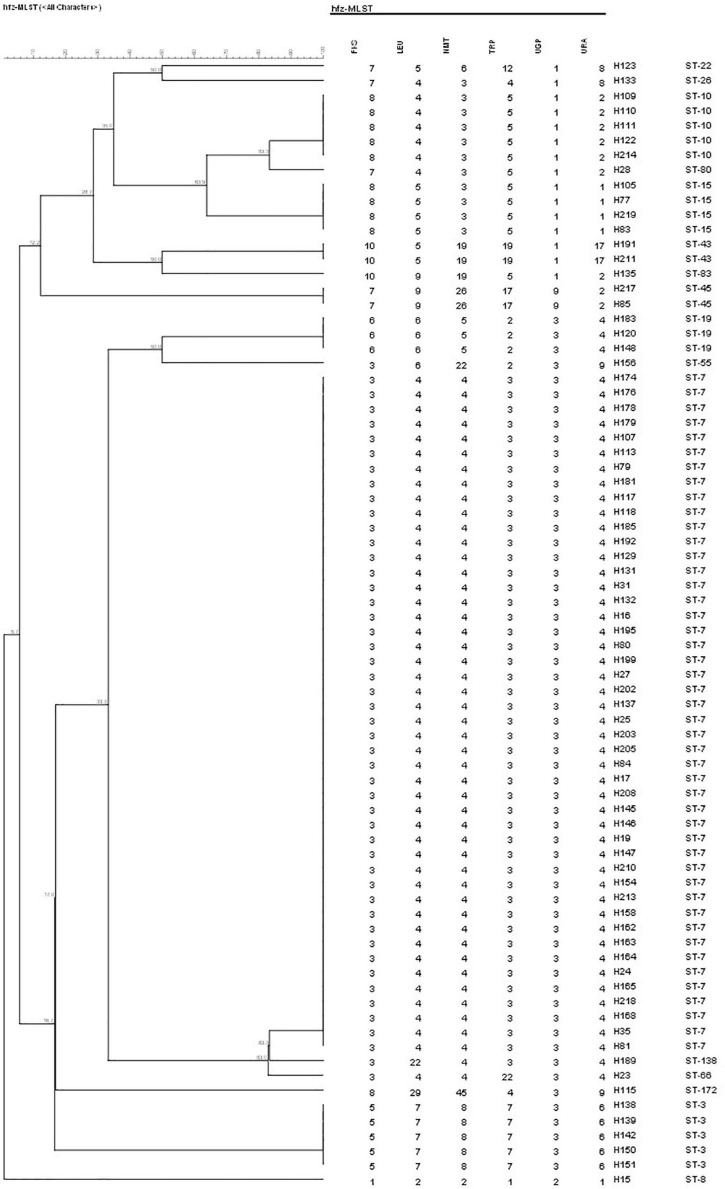
Unweighted pair group method with arithmetic means dendrogram *via* BioNumerics software (version 7.6) showing the similarities among *C. glabrata* isolates. A similarity exceeding 80% was considered clonally related. The dendrogram demonstrates the genotyping of 76 vaginal *C. glabrata* isolates collected in the Obstetrics and Gynecology Hospital of Fudan University. Sequence similarity was observed between ST10 and ST80 (83.3%), ST7 and ST138 (83.3%), and ST7 and ST66 (83.1%).

**FIGURE 2:**
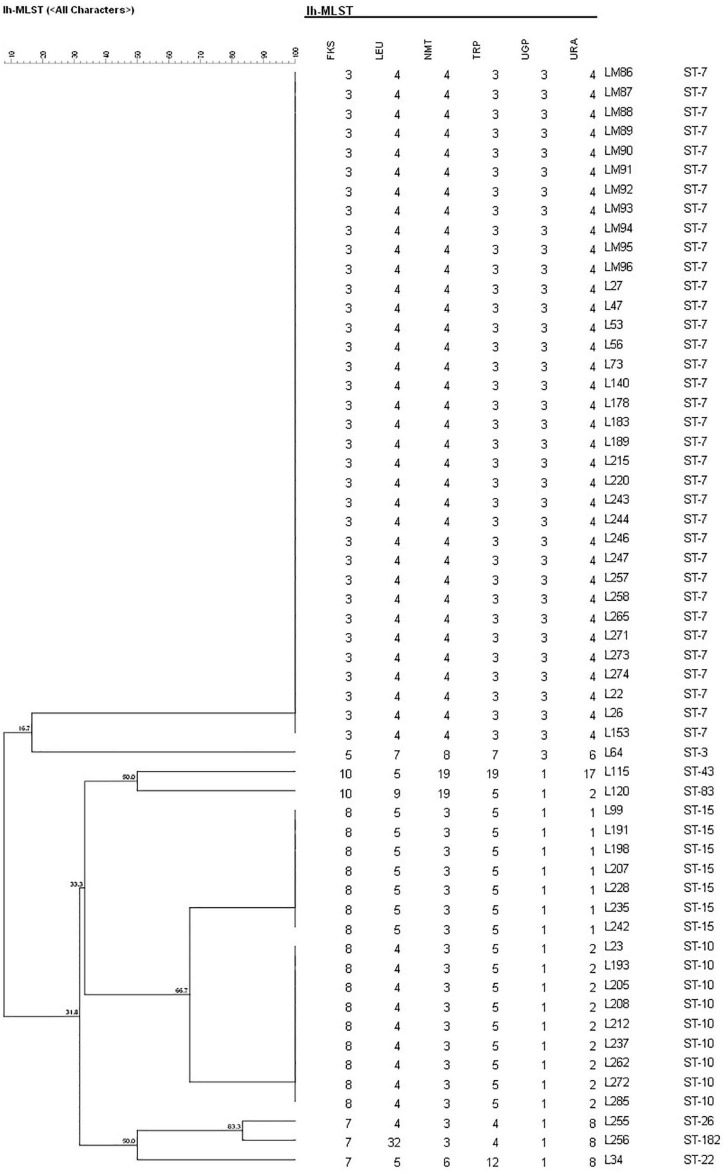
Unweighted pair group method with arithmetic means dendrogram *via* BioNumerics software (version 7.6) showing the similarities among *C. glabrata* isolates. A similarity exceeding 80% was considered clonally related. The dendrogram demonstrates the genotyping of 57 non-vaginal *C. glabrata* isolates in Longhua Hospital, Shanghai University of Traditional Chinese Medicine. Sequence similarity was observed between ST26 and ST182 (83.3%).

A total of 17 different STs were identified using MLST in 133 *C. glabrata* isolates. ST7, which accounted for the largest proportion (81/133, 60.9%), was the major ST type, followed by ST10 (14/133, 10.5%), ST15 (11/133, 8.3%), and ST3 (6/133, 4.5%). The remaining genotypes contained 3 isolates per ST19 and ST43 and 2 isolates per ST22, ST26, ST45, and ST83. The remaining 7 STs (ST8, ST55, ST66, ST80, ST138, ST172, and ST182) contained one isolate each. Moreover, the vaginal *C. glabrata* strains showed a greater genetic diversity compared with that of the non-vaginal strains (Table 3). An unrooted dendrogram using a categorical value was then constructed to define the genetic relatedness of the isolates. As shown in Figures 1, 2, sequence similarity (>80%) was observed between ST26 and ST182 (83.3%), ST10 and ST80 (83.3%), ST7 and ST138 (83.3%), and ST7 and ST66 (83.1%). The other STs showed a high genetic diversity.

**TABLE 3:** Summary of multilocus sequence typing genotypes for the 76 vaginal and 57 non-vaginal *C. glabrata* isolates.

Strains	Genotype	Isolates (*n*)
Vaginal *C. glabrata* (*n* = 76) (16 different genotypes)	ST7	46
	ST3, ST10	5
	ST15	4
	ST19	3
	ST43, ST45	2
	ST8, ST22, ST26, ST55, ST66, ST80, ST83, ST138, ST172	1
Non-vaginal *C. glabrata* (*n* = 57) (9 different genotypes)	ST7	35
	ST10	9
	ST15	7
	ST3, ST22, ST26, ST43, ST83, ST182	1

### Multilocus Sequence Typing Genotyping and Antifungal Susceptibility

**FIGURE 3:**
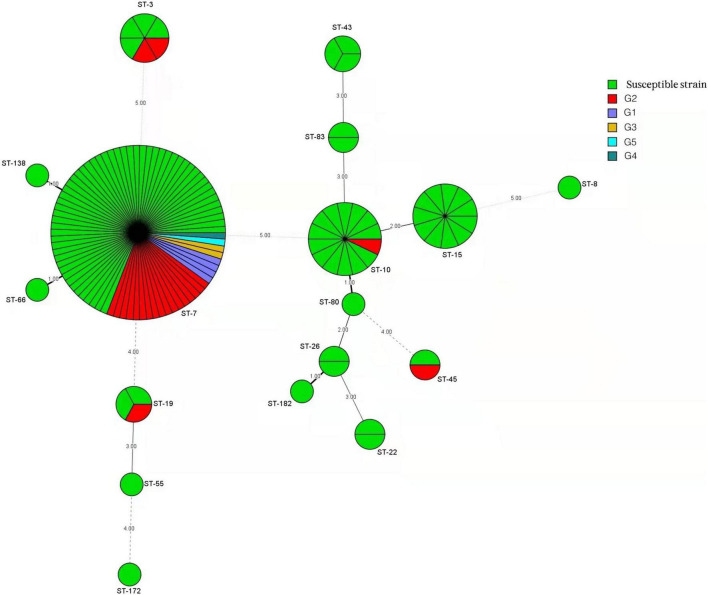
Minimum spanning tree conducted by six housekeeping gene loci of 133 *C. glabrata* isolates *via* the BioNumerics software (version 7.6) showing the relationship between the sequence types (STs) and susceptibility patterns of five antifungals tested (fluconazole, itraconazole, voriconazole, amphotericin B, and caspofungin). Each circle corresponds to a ST genotype. The green circles represent a susceptible phenotype, and the other five differently colored circles represent phenotypes comprised of group 1 (G1) to group 5 (G5). The circle size corresponds to the isolate number. Dark, dashed, and thin lines surrounding ST circles represent the genetic distance.

The minimum spanning tree analysis was used to determine the association between MLST genotype and antifungal resistance profiles (Figure 3). Of the 133 *C. glabrata* isolates, four isolates with a resistant phenotype to caspofungin all belonged to the ST7 genotype. The 10 non-vaginal fluconazole-resistant strains were ST7. The predominant ST7 genotype contained multiple antifungal resistance profiles (G1–G5) (Table 4). Four G1 isolates, 17 G2 isolates, 2 G3 isolates, 1 G4 isolate, and 1 G5 isolate belonged to the ST7 genotype. Additionally, 1 ST10 isolate, 1 ST19 isolate, 1 ST45 isolate, 2 ST3 isolates, and 23 ST7 isolates were resistant to fluconazole and itraconazole. One additional ST7 isolate was also resistant to voriconazole.

**TABLE 4:** Multilocus sequence typing (MLST) genotype and antifungal susceptibility clusters.

Antifungal susceptibility (FLC, ITC, VRC, CAS, AMB)	MLST type	Isolates (Number)
Susceptible (S, S, S, S, S)	ST7	56
	ST10	13
	ST15	11
	ST3	4
	ST43	3
	ST19, ST22, ST26, ST83	2
	ST8, ST45, ST55, ST66, ST80, ST138, ST172, ST182	1
G1 (R, R, R, R, S)	ST7	4
G2 (R, R, R, S, S)	ST7	17
	ST3	2
	ST10, ST19, ST45	1
G3 (S, R, R, S, S)	ST7	2
G4 (R, S, R, S, S)	ST7	1
G5 (R, S, S, S, S)	ST7	1

The resistant isolates showed resistance to at least one antifungal. Additionally, 25 out of 30 (83.3%) *C. glabrata* isolates with a resistant phenotype were correlated with the predominant genotype (ST7), while the rest belonged to ST3 (2 isolates), ST10 (1 isolate), ST19 (1 isolate), and ST45 (1 isolate). The percentages of resistant phenotypes were as follows: 7.1% (1/14), 33.3% (2/6), 33.3% (1/3), and 50% (1/2) in ST10, ST3, ST19, and ST45, respectively. ST7 was the dominant genotype in *C. glabrata* isolates with cross-resistance to azoles. The susceptible strains showed a greater genetic diversity compared with that of the resistant strains.

## Discussion

Fungal infections caused by *C. glabrata*, especially those associated with resistance to fluconazole, have resulted in a global health problem (Chen et al., 2021; Darwish et al., 2021; Rodrigues et al., 2021). Investigating the prevalence of *C. glabrata* molecular genotypes is important for preventing local epidemic spreading. Previous studies have shown a high variability in *C. glabrata* genotypes isolated from various sources (Diaz-Garcia et al., 2021). However, we do not have sufficient data yet on vaginal and non-vaginal *C. glabrata* isolates and their genotype profiles and antifungal resistance.

Numerous studies have shown reduced susceptibility to fluconazole or voriconazole among *C. glabrata* isolates (Song et al., 2020; Zeng et al., 2020, 2021), such as the large survey of *Candida* isolates from the Asia-Pacific region (Tan et al., 2016). A study examining 77 hospitals in China (across seven administrative regions) over a period of 3 years has shown an overall fluconazole resistance rate of 10.2% (3.1–11.4%) and a decrease in voriconazole susceptibility in *C. glabrata* (Xiao et al., 2020). Klotz et al. (2016) have also reported a high prevalence of azole resistance in *C.* glabrata, showing resistance rates of 25 and 30% to fluconazole and voriconazole, respectively.

In our present study, we collected 677 vaginal *C. glabrata* strains and 254 non-vaginal *C. glabrata* isolates. Antifungal susceptibility showed no statistical difference between the vaginal and non-vaginal *C. glabrata* isolates with respect to azole resistance (fluconazole, 4.1 vs. 5.9%, *p* = 0.25; itraconazole, 7.4 vs. 4.3%, *p* = 0.09; itraconazole, 3.8 vs. 5.1%, *p* = 0.39). These results agreed with those of Meletiadis et al. (2017), showing that the resistance rate in *C. glabrata* is only 2.3% for fluconazole and 1.7% for voriconazole, as determined using the eEUCAST method. In our present study, the resistance rates were much lower than those mentioned in the abovementioned studies, which may be explained by differences in methodology and sample sources. In other words, there is no strong resistance because resistance is biased by the strain selection. Additionally, overtreatment, including antifungal abuse in different regions of the world, may also contribute to reduced susceptibility.

A further analysis was conducted including 76 vaginal *C. glabrata* isolates obtained from patients with VVC and 57 non-vaginal isolates obtained from samples of urine, mouth swabs, sputum, blood, and fecal swabs. We found that, unlike vaginal *C. glabrata* isolates, which are mainly responsible for causing VVC in young women, non-vaginal *C. glabrata* commonly infected elderly patients with a mean age of 79.1 along with multiple underlying diseases. Our result was in accordance with the findings of other studies on non-vaginal *C. glabrata* as the most prevalent *Candida* species in elderly patients (Gupta et al., 2015).

With few exceptions, our results show that 26 isolates were cross-resistant to three azole antifungals, and the strains with fluconazole resistance were usually cross-resistant to other azole antifungals, such as itraconazole and voriconazole. These results agree with those obtained by Song et al. (2020) and Boonsilp et al. (2021). Conversely, fluconazole resistance was found in 30 of the 133 *C. glabrata* isolates examined in our present study. All of the strains exhibited susceptibility to amphotericin B, which was similar to the results obtained by Hou et al. (2017) in a large study conducted in China. Only four non-vaginal *C. glabrata* strains showed caspofungin resistance, which agrees with the results obtained in previous studies (Wiederhold, 2016). While the overall resistance to azoles, caspofungin, and amphotericin B remains rare, cross-resistance and multidrug resistance have emerged in *C. glabrata* strains, highlighting the importance of continued surveillance for antifungal susceptibility trends.

The molecular typing test included PFGE, microsatellite length polymorphism, and MLST. Among them, MLST is frequently used for strain phylogeny and global epidemiology. We used MLST to analyze the genetic relationships and increase the understanding of candidemia epidemiology. We examined the genetic diversity in *C. glabrata* isolates. The predominant ST7 genotype was observed in both vaginal and non-vaginal *C. glabrata* isolates. Conversely, vaginal *C. glabrata* strains showed a greater genetic diversity than that of non-vaginal strains, as assessed using MLST genotyping. Sequence similarity, assessed using a dendrogram, was only observed between ST26 and ST182, ST10 and ST80, ST7 and ST138, and ST7 and ST66. The other STs showed a high genetic diversity.

In line with our findings, several studies performed in Japan, South Korea, and China also indicated that ST7 is the major genotype associated with *C. glabrata* (Dodgson et al., 2003; Hou et al., 2017; Byun et al., 2018; Canela et al., 2021). Conversely, Boonsilp et al. (2021) have shown that the most common ST is ST55 (60%), followed by ST7 (20%), ST195 (10%), and ST199 (10%), among *C. glabrata* isolated from blood samples in Bangkok, Thailand. This genetic variation may stem from geographical bias, suggesting that the genetic variation among clinical isolates in a specific area cannot be extended to other areas.

Our analyses of ST genotypes and antifungal susceptibility profiles indicate that isolates belonging to the ST7 genotype possessed every pattern of resistance (G1–G5). Specifically, 25 out of 30 (83.3%) *C. glabrata* isolates with resistant phenotype belonged to the ST7 genotype. As shown in Figure 3, several identical STs (ST3, ST7, ST19, ST10, and ST45) were found to possess different antifungal profiles. Besides this, these antifungal profiles were either G2 (cross-resistant to fluconazole, itraconazole, and voriconazole) or susceptible phenotypes. The strains grouped in other STs identified in our present study (e.g., ST15, ST43, and ST22) all displayed a susceptibility pattern with no exceptions. Interestingly, four caspofungin-resistant non-vaginal isolates were all ST7. Our results are consistent with those obtained in previous studies. These studies revealed that the antifungal resistance profile shows no correlation with the predominant genotype in *C. glabrata* isolates (Klotz et al., 2016; Amanloo et al., 2018). However, in contrast with the results obtained in our present study, one study by Kiasat et al. (2019) demonstrated a positive association between *C. glabrata* population structures and the predominant genotype (GT27) with resistance phenotype to antifungal drugs. Dhieb et al. (2015) have shown similar results. This discrepancy may be due to differences in genotyping assays (microsatellite analysis) or sources of clinical isolates.

## Conclusion

In our present study, we explored the molecular epidemiology and antifungal susceptibility profiles of vaginal and non-vaginal *C. glabrata* isolates in two hospitals in China. Overall, our survey revealed cross-resistance in vaginal and non-vaginal *C. glabrata* isolates. The ST7 genotype was the predominant genotype and showed no correlation with the strain resistance profile.

## Data Availability Statement

The original contributions presented in the study are included in the article/Supplementary Material, further inquiries can be directed to the corresponding author/s.

## Ethics Statement

Written informed consent was obtained from the individual(s) for the publication of any potentially identifiable images or data included in this article.

## Author Contributions

CY and DY conceived the study. YC and YW performed the experiments, analyzed the results, and wrote the manuscript. KL assisted in MLST genotyping and antifungal susceptibility testing and modified the manuscript. All authors read and approved the final version of the manuscript.

## Conflict of Interest

The authors declare that the research was conducted in the absence of any commercial or financial relationships that could be construed as a potential conflict of interest.

## Publisher’s Note

All claims expressed in this article are solely those of the authors and do not necessarily represent those of their affiliated organizations, or those of the publisher, the editors and the reviewers. Any product that may be evaluated in this article, or claim that may be made by its manufacturer, is not guaranteed or endorsed by the publisher.
